# Epigenetic modifications in the accumulation and function of myeloid-derived suppressor cells

**DOI:** 10.3389/fimmu.2022.1016870

**Published:** 2022-11-11

**Authors:** Liangjie Xu, Cuicui Zhou, Yi Liang, Tinpan Fan, Fen Zhang, Xinxin Chen, Wei Yuan

**Affiliations:** Department of Cardiology, Affiliated Hospital of Jiangsu University, Zhenjiang, Jiangsu, China

**Keywords:** epigenetic modification, myeloid-derived suppressor cells, DNA methylation, histone acetylation, N6-methylation, ubiquitination

## Abstract

Myeloid-derived suppressor cells (MDSCs) are key players under various pathologic conditions, such as cancer. Epigenetic modifications such as DNA methylation, RNA-mediated processes, and histone modification can alter gene transcription, and thus regulating pathological process. Studies have shown that epigenetic modification contributes to the accumulation and function of MDSCs. This review summarizes the crosstalk between the epigenetic alterations and MDSCs functions, and briefly introduces how the accumulation and function of MDSCs caused by epigenetic modification impact on the disease development, which represents as a promising therapeutic strategy for the related disorders.

## Introduction

Myeloid-derived suppressor cells (MDSCs) are a group of immature myeloid cells that has been identified as a universal regulator of immune function under many pathologic conditions (1). MDSCs are characterized by the expression of CD11b^+^Gr-1^+^ in mice, and defined by the expression of Alpha M-Integrin CD11b and myeloid (CD14 and CD33) in human (2). MDSCs can be subdivided into two primary subtypes, polymorphonuclear MDSCs (PMN-MDSCs), and mononuclear MDSCs (M-MDSCs). The phenotype and morphological characteristics of PMN-MDSCs are similar to neutrophils, while M-MDSCs are similar to monocytes (3). Besides the two main populations, a small group (less than 3%) of MDSCs displays myeloid colony forming activity including myeloid progenitors and precursors (4). MDSCs inhibit the immune response by suppressing the proliferation of pro-inflammatory T cells and B cells, induce apoptosis and block proinflammatory cytokine expression in activated cells (5, 6). Immune suppression is the main function of MDSCs, and the main targets of MDSCs are T cells. MDSCs exert the immunosuppressive function through a variety of mechanisms and pathways. The main factors implicated in the immunosuppressive function of MDSCs include inducible nitric oxide synthase (iNOS), reactive oxygen species (ROS), Arg-1(Argininase-1), IL-10, Transforming growth factor beta (TGF-β), indoleamine 2,3-dioxygenase (IDO), cyclooxygenase-2 (COX-2), L-selectin, etc. Accumulation of MDSCs also involves many factors including STAT3, IRF8, C/EBPβ, Notch, adenosine receptors A2b signaling, NLRP3, retinoblastoma protein 1 (Rb1) and so on (7). MDSCs play dual roles in different type of biological processes and diseases. The intrinsic functions of MDSCs are maintaining immunotolerance, protecting from over-reactive responses, homeostasis, and promoting tissue repair in immunoregulation, angiogenesis, and wound healing. However, the role of MDSCs shift from protective to harmful when the exposure to unresolved inflammatory triggers prolongs, which stimulated excessive the accumulation of MDSCs, leading to unbalanced immune homeostasis and dysfunctional immune response. These effects eventually result in expanded the inflammatory response, the formation of immunosuppressive microenvironment, tissue damage, and angiogenesis (8).

Interestingly, several key findings shows that epigenetic modifications regulate the accumulation and immunosuppressive function of MDSCs, such as DNA methylation at CpG islands in promoter regions, histone acetylation or methylation, N6 -methylation of adenosine (m6 A) in mRNA, microRNAs (miRNA), and long non-coding RNAs (lncRNAs), etc. Epigenetic modification of MDSC’s functional plasticity leads to the remodeling of its characteristics, therefore reframing the microenvironment towards countering tumor growth and metastasis (9). For example, MDSC – associated miRNA shapes MDSCs development, differentiation and activation which impacts the development of tumor, and resistance to treatment with immune checkpoint inhibitors (8). In this review we will discuss the relationship between epigenetic modifications and the accumulation and function of MDSCs in various biological processes and diseases.

## Epigenetic modification in MDSCs

### Role of miRNA

miRNAs are short non-coding RNAs which regulate gene expression through deadenylation and translational suppression of target mRNAs. A large number of miRNAs have been found to play key roles in inflammation, cancer, infection, and autoimmune disease. MDSC-associated miRNAs (miR-146a, miR-155, miR-100, miR-125a, miR-125b, miR-99b, miR-146b and let-7e) are enriched in correlation with myeloid cell accrual and contribute to the immunotherapy resistance in melanoma patients (10). In tumor environment, the miR-449c/STAT6 axis is associated with the expansion of M-MDSCs, thus, blocking miR-449c axis may offer an effective epigenetic therapy to inhibit tumor progression (11). Sepsis is a dysregulated host response to infection characterized by MDSCs infiltrations in circulation, which leads to the life-threatening organ dysfunction (12, 13). MDSCs miRNA, such as miR-21-5p, miR-181a, miR-106a, and miR-17-5p, expression patterns are different according to sepsis severity, outcome, and different time points after sepsis, exhibiting persistent and chronic immune suppression in sepsis survivors (14). miR-214-3p regulates the immunosuppressive function and differentiation of M-MDSCs by *Olfr29-ps1* which is a lncRNA pseudogene (15).It has been described that a wide variety of miRNA can contribute to the differentiation and function of MDSCs by three crucial signaling pathways: Ras/Raf/MAPK, STAT, and the PI3 kinase signaling pathways (16). MDSC-related miRNA could also modulate the activity of other signaling pathways, stimulating mechanistic target of rapamycin (mTOR) and protein kinase B (Akt) triggering phosphatase and tensin homolog deleted on chromosome 10 (PTEN), leading to the accumulation of tumor- derived MDSCs (8).

### Role of DNA methylation

DNA Methylation is a major epigenetic modification and dependents on DNA methyltransferases (DNMTs); it usually results in reversible gene silencing (17, 18). DNMT1 binds preferentially to hemimethylated DNA and is crucial for cell maintenance (19). The DNMT3 family, including 3a and 3b, is necessary for *de novo* methylation (19). Δ9-tetrahydrocannabinols (THC) is an exogenous cannabinoid derived from the cannabis sativa plant, and it could induce the accumulation of immunosuppressive MDSCs in mice (20). THC treatment of MDSCs could inhibit the expression of DNMT3a and DNMT3b. Signal transducer and activator of transcription 3 (STAT3) is a key transcription factor to mediate the accumulation, activation and function of MDSCs (21–23). Several new findings suggest that STAT3 is a crucial transcription factor for DNA methyltransferase and histone methyltransferase to induce an epigenetic silencing program in MDSCs (24–27). Additionally, the methylation at the promoter region of Arg1 and STAT3 are decreased in THC-induced MDSCs, leading to the secretion of STAT3-related cytokines, such as IL-6 and IL-10, which then activates STAT3 phosphorylation. The phosphorylated STAT3 can bind to the promoter region of Arg1 and S100A8 and induce the overexpression of Arg1 and S100A8, which enhance the immunosuppressive function of MDSCs (28, 29).

The DNA methyltransferase inhibitor decitabine (DAC) reduces MDSCs accumulation and improves antigen-specific CTL activation. DAC treatment stimulates the Irf8 promoter demethylation and expand IRF8^+^MDSCs in tumor microenvironment. Since interferon regulatory factor 8 (IRF8) is a key regulator of myeloid cell lineage differentiation through promoter DNA methylation (30, 31), the above finding implies that DNA methylation mediates MDSCs accumulation and post-MDSCs lineage differentiation. TNFα can induce MDSCs cell death in a dose-and RIP1-dependent manner. The promoters of Tnf, Ripk1, and Ripk3 are hypermethylated in tumor-induced MDSCs. MDSCs depend on the IL6-STAT3-DNMT-TNFα-RIP1 pathway to maintain MDSCs survival and accumulation (32). The activation of MDSCs is responsible for Aldehyde dehydrogenase 1 (ALDH1)- related radiation resistance in oral squamous cell carcinoma (OSCC). In addition, ALDH1 is positively associated with DNMT3. Hypomethylating agents decrease the radiation resistance of ALDH1-positive cancer cells, and attenuate the activation of MDSCs in OSCC (33). The elevated expression of DNMT1 compensates the downregulation of DNMT3a in CD33^+^HLA-DR^-^ cells. Ten-eleven translocation (TET) enzymes upregulates the expressions of transforming growth factor-β1 (TGF-β1), galectin-9, T-cell immunoglobulin and mucin-domain containing-3 (TIM-3) and Arg1 by inducing demethylation of their promotors in CD33^+^HLA-DR^-^ cells (34). In colorectal cancer (CRC) patients, DNA methylation-related genes are upregulated in tumor-infiltrating I-MDSCs, but are downregulated in tumor-infiltrating PMN-MDSCs (35). Not only the MDSCs derived from co-cultures with ovarian and colorectal cancer cells, but also the primary MDSCs from ovarian carcinoma patients show MDSC-specific hypermethylation signature. Prostaglandin-E2 (PGE2) upregulates DNA methyltransferase 3a (DNMT3a) in MDSCs which leads to MDSC-specific hypermethylation signature and immunosuppressive phenotype (36). There are a set of 189 differentially methylated CpGs within 10 CpG loci exhibiting consistent differential methylation states between gMDSC and neutrophils from adult and neonatal sources. *FATS, YAP1, VCL, KREMEN2, MCC-1, UBTF*, and *EFCC1* are associated with these loci and involved in immune responses (37).

### Role of histone modifications

Histone acetylation regulates gene transcription through histone deacetylase (HDAC) or histone acetyl transferase (HAT), which deacetylates or acetylates the lysine residues in the histone. This process of chromatin modification does not involve DNA binding elements (36). HDAC11 is a negative regulator of anti-inflammatory cytokine IL-10, which is secreted in response to pro-inflammatory signal by virtually all immune cells, such as T cells, B cells, macrophages, myeloid cells, etc (38–41). The MDSCs in HDAC11 deficient mice shows a more suppressive function. Furthermore, the deficiency of HDAC11 gene reduces the level of IL-10 secretion, indicating that HDAC11 can regulate the formation and suppressive phenotype of MDSCs (42). Elevated levels of immature myeloid-derived suppressor cells (I-MDSCs) and PMN-MDSCs are observed in colorectal cancer (CRC) tissues. In CRC patients, HDAC–related genes are upregulated in tumor-infiltrating I-MDSCs, while HAT-related genes are downregulated. In contrast, HDAC-related genes are downregulated in tumor-infiltrating PMN-MDSCs. HDAC inhibitor downregulates the expression of Arg1, monocyte chemokine receptor (CCR2), and ITGAL, which contributes to the immunosuppressive function and myeloid cell chemotaxis (35). All above evidence shows the importance of HDAC activation in mediating MDSCs suppressive function and chemotaxis.

iNOS is a crucial mediator of suppressive function of mo-MDSCs; however, the epigenetic regulation iNOS expression involved in MDSCs function is still unclearly. Histone methyltransferase SETD1B mediates trimethylation of histone H3 lysine4 (H3K4Me3) at the nos2 promoter to stimulate iNOS expression in tumor-derived MDSCs. This process, bypasses the common pathway of iNOS activation in myeloid cells, which is mediated by the upregulation of IRF8, an essential transcriptional activator of iNOS (43). Osteopontin (OPN) is closely correlated with poor prognosis in MDSCs involved human pancreatic carcinoma. OPN is primarily expressed in mo-MDSCs, whereas programmed death receptor ligand-1 (PD-L1) is mainly expressed in PMN-MDSCs, which might be the reason that the immune checkpoint inhibitor (ICI) therapy failed in these patients. The WDR5-H3K4me3 epigenetic axis suppresses pancreatic tumor immune escape by blocking OPN expression in mo-MDSCs, which further improves the efficacy of ICI therapy targeting PD-L1 (44). Under physiological condition, monocyte normally differentiates into dendritic cells (DCs) and macrophages. But in cancer, monocyte preferentially differentiates to PMN-MDSC through epigenetic silencing of the retinoblastoma (Rb) protein gene mediated by histone deacetylase 2 (HDAC-2) (45). The lncRNA, HOXA transcript antisense RNA myeloid-specific 1 (Hotairm1), promotes myeloid precursor to differentiate to MDSCs in sepsis (46). Histone demethylase KDM6A inhibits H3K27 trimethylation that is necessary for PU.1 binding at Hotairm1, and induces Hotairm1 transcription activation in MDSCs during sepsis (47). Therefore, the epigenetic modification of transcription factor PU.1 in MDSCs may be a potential immune-checkpoint therapy target for sepsis.

### Role of N6 -methylation

N6 -methyladenosine (m6 A) modification has been observed on almost every type of RNAs. Nearly all m6A sites were identified on the consensus motif RRACH (R=A/G, H=A/C/U) (43), which are enriched around the 3’UTR, near the stop codons (48–50). m6A methylation is modified by the writers, including METTL3/14/16, WTAP, ZC3H3, CBLL1, VIRMA, RBM15/15B, and KIAA1429, and removed by the erasers, such as FTO and ALKBH5. The m6A-binding proteins are recognized as reader proteins, including YTHDF1/2/3, IGF2BP1/2/3, YTHDC1/2, and HNRNPC/A2B1 (51, 52). m6A modification regulates miRNA biogenesis, m6A switch, XIST-dependent X chromosome inactivation, pre-mRNA splicing, RNA translocation, RNA translation, RNA stability, and RNA decay (53).

ALKBH5 regulates m^6^A density and splicing event during ICI treatment in the TME (tumor microenvironment). The number of polymorphonuclear MDSCs (PMN-MDSCs) are decreased in Alkbh5-KO tumors and MDSCs depletion attenuates tumor growth, which suggest that the regulation of immunosuppressive MDSCs recruitment *via* Alkbh5 is indispensable during GVAX/anti-PD-1 therapy. ALKBH5 targets Mct4 to suppress MDSCs expansion and lactate levels which directly affects the recruitment of MDSCs in tumor sites. ALK-04, a specific inhibitor of ALKBH5, improves the efficacy of anti-PD-1 treatment in melanoma (54, 55). *Olfr29-ps1*, a lncRNA pseudogene, is expressed in MDSCs and is upregulated by IL-6. *Olfr29-ps1* induces immunosuppressive function and differentiation of M-MDSCs through a METTL3-modified *Olfr29-ps1*/miR-214-3P/MyD88 axis (15). The level of METTL3 is positively correlated with CD33^+^MDSCs in cervical cancer (CC) patients, and both METTL3 and CD33^+^MDSCs are independent prognostic factors for CC. The recruitment of CD33^+^MDSCs and tumor-derived MDSCs are attenuated by inhibiting METTL3 *in vitro* (56). Cisplatin inhibits tumor proliferation and metastasis in patients with bladder cancer (BC) by blocking the accumulation and immunosuppressive function of fibrocytic-MDSCs(f-MDSCs) in BC tissue. In addition, Cisplatin regulates f-MDSCs by inhibiting the methylation of granulocyte colony-stimulating factors (G-CSF) *via* targeting METTL3 (15).

### Role of ubiquitination

Ubiquitination is a type of post-translational modifications that is essential for multiple cellular processes (57). Three enzymes forms of the ubiquitination system: E1 ubiquitin-activating enzyme, E2 ubiquitin-conjugating enzyme, and E3ubiquitin ligase (58). STAT3 also plays a crucial role in the ubiquitination process. Tumor necrosis factor receptor-associated factor 6 (TRAF6) is an E3 ubiquitin ligase that induces the polyubiquitination of target proteins. TRAF6 is highly expressed in MDSCs from both lung cancer patients and tumor-bearing mice. Knockdown of TRAF6 attenuates the immunosuppressive function of MDSCs. Moreover, TRAF6 improve the immunosuppression of MDSCs by promoting K63-linked polyubiquitination and phosphorylation of STAT3 (59, 60). P66a (Gatad2a) is a protein involved in epigenetic regulation. It could mediate the K63 ubiquitination and Y705 phosphorylation of STAT3 to impact on MDSCs activation. In addition, p66a has a negative effect on the differentiation and immunosuppressive function of MDSCs (GM-CSF plus IL-6 treatment) (61). It has been shown that TRAF6 can interact with STAT3 in MDSCs, implying that p66a may inhibit K63-binding ubiquitination of STAT3 through inhibiting the interaction between STAT3 and TRAF6 (61).

Downregulation of Ube2o in alveolar macrophages (AMs) triggers TAK1- NF-κB/ERK/JNK signaling and CXCL10-induced CCL12 expression by inducing TRAF6 polyubiquitination and blocking DDX3X degradation in tumor environment, resulting in mo-MDSCs recruitment through the CXCL1-CXCR3/TLR4-CCL12 axis (62). Cullin 4B (CUL4B), which is a scaffold protein of Cullin 4B-RING E3 ligase complex (CRL4B), acts as a negative regulator of MDSCs functions through several epigenetic mechanisms in various cancers. CRL4B-induced H2AK119 monoubiquitination is important for epigenetic inactivation of tumor suppressors *via* SUV39H1/HP1/DNMT, PRC2, and SIN3A/HDAC complexes (63–65). Deficiency of CUL4B in hematopoietic system simulates the accumulation and activity of MDSCs through AKT/β-catenin pathway, thus inhibiting the formation of tumor-permissive microenvironment (66). IL-6 secreted from CUL4B-dificient MDSCs enhances the stem cell-like properties in cancer cells through IL-6/STAT3 pathway (67).

### Role of acetylation

Acetylation is linked to gene expression by opening up the chromatin for appropriate transcriptional machineries to access the DNA epigenetic modification (68). Lysine acetyltransferase 6A (KATA6) is a member of the MYST-family of histone acetyltransferases (HATs) and functions as a chromatin modifier with acetyltransferase activity to acetylate histone and nonhistone proteins (69). Phosphorylated SMAD3 involved in MDSCs recruitment, which promotes metastasis by binging to CXCL2, IL-6 (70, 71). KAT6A -acetylated SMAD3 upregulates immune response-related cytokine expression (IL-6, IL-22, and TNFα), and induces MDSCs recruitment that implies poor prognosis in triple-negative breast cancer (TNBC) (72).

## Epigenetic therapy targeting MDSCs

As the importance and functional roles of MDSCs in cancer and pathological conditions have been increasingly recognized, it is critical to examine how epigenetic therapy can impact on MDSCs and disease progression.

The combination epigenetic therapies can reverse tumor immune evasion and regulate T cells exhaustion state. For example, DNA-demethylating agents (DNA methyltransferase inhibitors [DNMTis]) in combination with histone deacetylase inhibitors (HDACis) can improve the efficacy of cancer immune therapy. This is due to the DNA-demethylating agents’ effect of altering immune response, activating silenced tumor suppressor genes, epigenetically reprograming tumor cells, and the histone deacetylase inhibitors’ effect of increasing the expression of abnormally silenced genes, which are downregulated by promoter DNA methylation (73). Michael et al. found that azacytidine (Aza) in combination with ITF-2357 could stimulate IFNα/β pathway related genes and suppress MYC signaling to reverse immune evasion and activate T cell phenotypes in non-small-cell lung cancer (NSCLC) patients, indicating that the combination of epigenetic treatments can have a potent anti-tumor effect (74). Low dose of DNA methyltransferase and histone deacetylase inhibitors, such as Aza and entinostant, had limited effect on the proliferation and/or apoptosis of HNM007, 4T1, LLC1 and CD45.1 donor cells; however, they could impede MDSCs accumulation and decrease niche-promoting molecules in the premetastatic lung of pulmonary metastatic murine models. LD-AET treatment could suppress both M-MDSCs and PMN-MDSCs migration from bone marrow to the microenvironment by inhibiting the expression of CCR2 in MDSCs. Moreover, LD-AET induced MDSCs showed a more interstitial macrophage-like phenotype, and the patient overall survival was better under LD-AET treatment (75).

Epigenetic therapy can inhibit the immunosuppressive function of MDSCs and affect the disease prognosis. Cannabinoids have been showed to induce MDSCs through mediating the abundance and function of MDSCs (20, 76). Interestingly, the regulation of Cannabinoids on MDSCs mediated by several epigenetic alterations, including DNA methylation and RNA interference, which attenuate inflammation (77). For example, the CBD treatment of MDSCs reduce neuroinflammation in experimental autoimmune encephalomyelitis (EAE) mice. Thus, Cannabinoids treatment may play a key role in regulating inflammation through epigenetic pathways (78).

Immune-checkpoint inhibition (ICI) therapy is a revolutionary treatment for cancer. However, ICI therapy is less effective in cancers with complex immunosuppressive TMEs such as pancreatic and breast cancers. In these cases, the epigenetic therapy can improve the efficacy of ICI immunotherapy. DNA hypomethylating agents attenuated the radio-resistance of ALDH1-positive tumors by reducing ALDH1 and increasing DNA damages. Additionally, the activation of MDSCs and PD-L1 expression were significantly decreased by epigenetic therapy (33). The WDR5-H3K4me3 epigenetic axis can inhibit OPN expression, which can be used to compensate the PD-L1 ICI immunotherapy to obtain better efficacy in suppressing pancreatic tumor (44). In breast cancer mouse model, blocking KAT6A using WM-1119 inhibitor could significantly decrease MDSCs recruitment and CD4^+^/CD8^+^ T cells depletion; when WM-1119 and anti-PD-L1 antibody treatment were combined, the efficacy of MDSCs inhibition recruitment and CD4^+^/CD8^+^ T cells activation was better (72). Therefore, combining WM-1119 and anti-PD-L1 antibody treatment might be an efficient therapy for breast cancer patient with metastasis. Several HDAC inhibitors such as Trichostatin(TSA), Valproic acid, Entinostat, Ricolinostat, Mocetinostat, Sodium butyrate, Vorinostat, ACY241, and CG-745, could downregulate MDSCs immunosuppressive function and accumulation by inhibiting the activation and function of T cells, thus inhibiting tumor progression. In addition, HDAC inhibitor can increase the expression of PD-1 or PD-L1 on MDSCs, and therefore is a promising approach to overcome the limitations of cancer immunotherapy (79). Entinostat (ENT) is a histone deacetylase inhibitor. ENT+ICIs therapy prevents the recruitment of MDSCs into TME, inhibits the suppressive function of G-MDSCs, and improves tumor-free survival in pancreatic and breast cancer mouse models (80). Therefore, ENT+ICIs therapy is a new method to sensitize nonimmunogenic tumors, including pancreatic and breast cancers. ALKBH5 can sensitize tumors to anti-PD-1 therapy by regulating lactate and MDSCs accumulation in the TME. The vaccines that inhibit the immunosuppressive function of MDSCs display significant antitumor activities. When a vaccine contains both PD-L1-silencing shRNA and IL-12, it can exhibit anti-melanoma activities, because PD-L1-silencing shRNA can increase the T cell numbers, and IL-12can inhibit tumor growth (81).

## Concluding remarks

**Figure 1:**
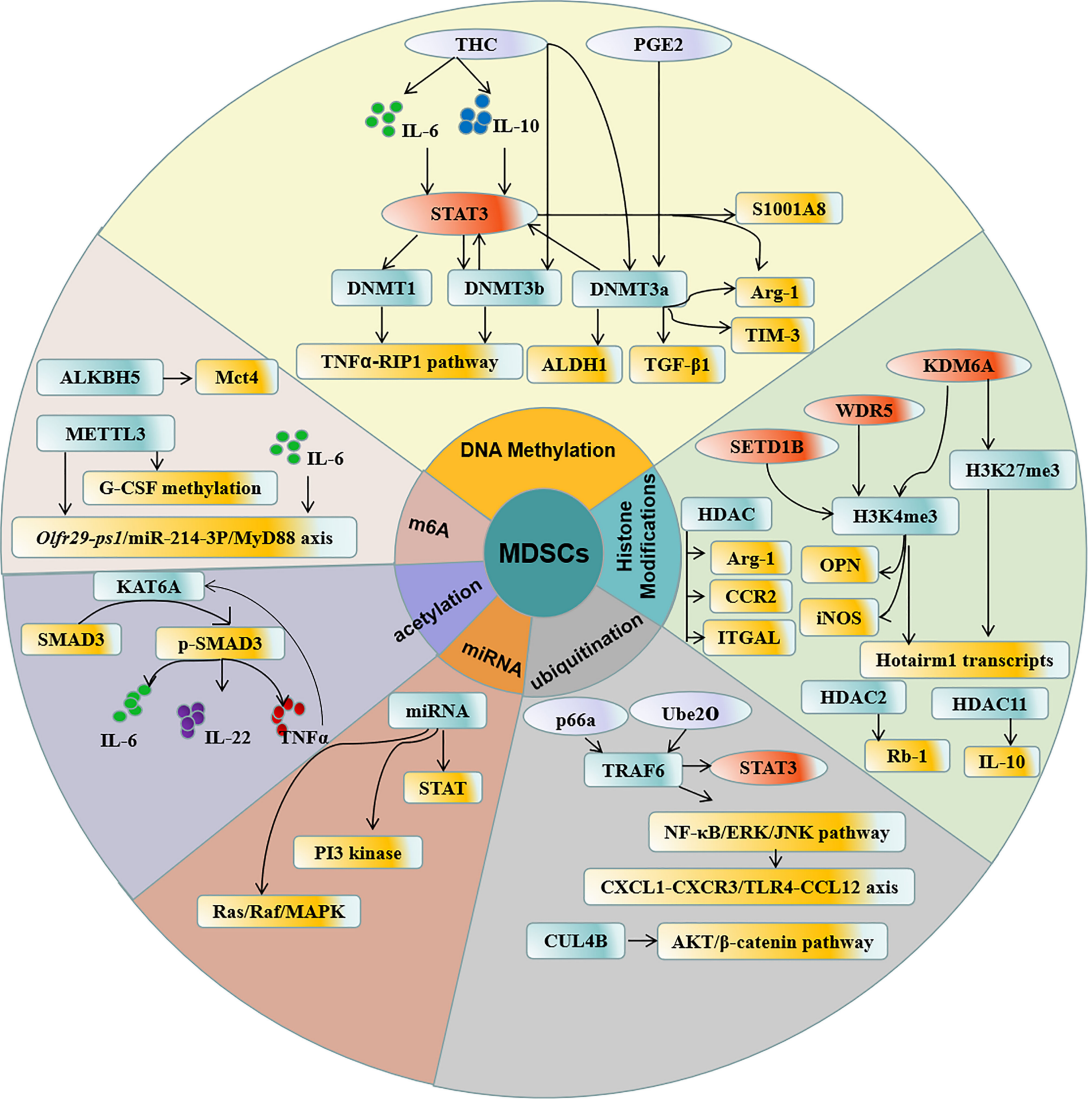
Regulation of epigenetic modification in MDSCs. DNA methylation, Histone acetylation, N6 -methylation, ubiquitination, acetylation, and miRNA participate in the epigenetic modification in MDSCs which contribute to the accumulation and function of MDSCs.

The epigenetic alterations of MDSCs are critical for a wide range of pathologic conditions (**Figure 1**), especially in the development of cancer and autoimmune disease. Specific changes in the epigenetic modification of MDSCs could be a potential marker to evaluate the stage and outcome of various diseases. In addition, better understanding the mechanism of MDSCs signaling pathways and activities regulated by epigenetic modification can provide more scientific basis for developing novel therapies to treat diseases.

## Author contributions

All authors listed have made a substantial, direct, and intellectual contribution to the work and approved it for publication.

## Funding

This work was supported by the medical research project of Jiangsu provincial health committee [NO. H2019075], and the Zhenjiang Cardiovascular Clinical Research Center Project [SS2018008].

## Conflict of interest

The authors declare that the research was conducted in the absence of any commercial or financial relationships that could be construed as a potential conflict of interest.

The reviewer JW declared a shared affiliation with the authors to the handling editor at the time of the review.

## Publisher’s note

All claims expressed in this article are solely those of the authors and do not necessarily represent those of their affiliated organizations, or those of the publisher, the editors and the reviewers. Any product that may be evaluated in this article, or claim that may be made by its manufacturer, is not guaranteed or endorsed by the publisher.
